# Three-Dimensional PET Imaging Reveals Canal-like Networks for Amyloid Beta Clearance to the Peripheral Lymphatic System

**DOI:** 10.3390/cells14221754

**Published:** 2025-11-10

**Authors:** Giselle Shim, Rudolf Hall, Zeming Zhang, Ibrahim M. Shokry, Alexandra To, Lillian Cruz, Mary C. Adam, Howard Prentice, Jang-Yen Wu, Hongbo Su, Rui Tao, for the Alzheimer’s Disease Neuroimaging Initiative

**Affiliations:** 1 Charles E. Schmidt College of Medicine, Florida Atlantic University, Boca Raton, FL 33431, USA; gshim2017@fau.edu (G.S.); rhall2017@fau.edu (R.H.); hprentic@health.fau.edu (H.P.); jwu@health.fau.edu (J.-Y.W.); 2 School of Veterinary Medicine, Ross University, Basseterre P.O. Box 334, Saint Kitts and Nevis; 3 Department of Civil, Environmental and Geomatics Engineering, Florida Atlantic University, Boca Raton, FL 33431, USA; suh@fau.edu

**Keywords:** amyloid beta, Aβ, artificial intelligence, AI, cervical lymph nodes, cLNs, convolutional neural network, CNN, lymphatic system, florbetapir, AV45, positron emission tomography, PET

## Abstract

**Highlights:**

**What are the main findings?**

- AI-enhanced 3D imaging is clearer than the original PET;
- Aβ is confined within canal-like compartmental networks in the brain;
- Those networks connect to the peripheral lymphatic system, facilitating Aβ clearance.

**What are the implications of the main finding?**

- Three-dimensional imaging offers new insights into mechanisms of Aβ clearance;
- It reveals the relationship between intracranial and extracranial Aβ signals;
- It suggests the existence of multiple pathways for Aβ efflux from the brain.

**Abstract:**

^18^F-Florbetapir PET imaging is widely used to assess amyloid-β (Aβ) burden in the brain, particularly in the context of Alzheimer’s disease (AD). Conventional assessments typically rely on selected individual slices, which may limit spatial accuracy and are prone to image blurring. In the present study, we introduce novel techniques to enhance the spatial resolution and clarity of Aβ signal visualization in individuals pretreated with ^18^F-florbetapir. PET scans were retrospectively obtained from the Imaging and Data Archive for twelve individuals, including six cognitively unimpaired subjects and six diagnosed with AD. Each dataset consisted of 346 raw images, comprising 90 axial, 128 coronal, and 128 sagittal slices. Images were reconstructed into a single 3D volume using the 3D Slicer platform. Crucially, we applied artificial intelligence or AI-driven signal enhancement techniques to suppress background noise and amplify Aβ signals. This AI-enhanced processing improved image clarity and enabled visualization of subtle and spatially organized signal patterns. To verify anatomical location, Aβ PET signals were registered with MRI. This integrated workflow allowed us to visualize Aβ signals across regions of interest, including the brain parenchyma, skull, and cervical tissues. Our analytical approaches revealed that Aβ signals are highly concentrated and confined within non-CNS fluid compartments, forming canal-like networks that extend from the brain parenchyma toward the skull base, particularly the occipital clivus, and connect to the cervical lymph nodes. Additional Aβ signals were observed along the internal carotid plexus. These findings suggest that, when reconstruction in 3D and enhanced with AI, ^18^F-florbetapir PET imaging may not only reflect Aβ plaque burden in the brain but also visualize soluble Aβ species concentrated within anatomical clearance pathways leading to the peripheral lymphatic system. This approach offers a new dimension to PET signal interpretation and highlights the potential of AI-enhanced 3D in advancing neuroimaging analysis.

## 1. Introduction

Amyloid beta (Aβ) accumulation is recognized as an initiating risk factor in the pathogenesis of Alzheimer’s disease (AD). The progression from early Aβ deposition to the onset of clinical symptoms typically spans many years, often extending over several decades [1]. This gradual buildup, commonly referred to as amyloid burden, can be non-invasively assessed using positron emission tomography (PET) imaging. To date, the U.S. Food and Drug Administration (FDA) has approved three fluorine-18-labeled radioligands for Aβ imaging: florbetapir, florbetaben, and flutemetamol. PET imaging is acquired slice by slice, with each slice typically 2.0 mm thick. Positron decay during scanning is corrected through post-processing algorithms. Despite its utility, PET imaging is inherently limited by spatial blurring, which complicates the direct interpretation of raw images. To standardize quantification, standardized uptake value ratios (SUVr) and Centiloid (CL) values are calculated from selected slices to represent Aβ levels in the brain. However, even with these metrics, discrepancies between image-based estimations and clinical diagnoses are apparent as high as 10–24% [2,3], underscoring the need for improved imaging and analytical approaches.

Multiple Aβ species exist in the brain. At the cleavage site, Aβ is a monomer with no known toxic potential [4,5], and radiotracers appear to lack affinity for these monomers [6]. Aβ peptides possess the unique ability to oligomerize into macromolecular structures, which are associated with neurotoxicity. These macromolecules may be either soluble or insoluble. Insoluble Aβ isoforms are deposited, and then become plaques that appear as sporadic clusters spread in the brain, visible under microscopic examination [7]. Unlike monomers, plaques have β-sheet structures, which are believed to be the binding targets of radioligands, with affinities reaching nanomolar levels [Kd = 3.7 nM; [8,9]]. Consequently, β-sheet interactions have become a central paradigm for interpreting PET signals as indicative of amyloid deposition and associated toxicity. In this context, extracellular plaques located in the interstitial fluid (ISF) compartment have been considered central to Aβ toxicity, forming the basis of the amyloid cascade hypothesis [10,11].

Recent research has shifted focus toward soluble Aβ oligomers, now considered the primary neurotoxic species driving AD progression. Experimental evidence indicates that micromolar concentrations of Aβ are sufficient to promote oligomerization [12] and β-sheet formation in the soluble phase [13]. Moreover, emerging data suggest that radioligands may bind not only to plaques but also to soluble oligomers. For example, studies of PiB, a prototype of florbetapir, indicate potential affinity for soluble Aβ oligomers [6]. These findings challenge the traditional plaque-centric view and highlight the need for imaging approaches capable of capturing both plaques and soluble Aβ species.

The goal of the present study is to better understand the nature of Aβ species that bind PET radioligands and are visualized in PET imaging. We hypothesize that observed Aβ signals represent soluble species that are concentrationally confined within non-CNS fluid compartments. These compartments may reflect clearance pathways from the brain to peripheral anatomies, specifically the peripheral lymphatic system. To test this hypothesis, we employed multi-pronged approaches to address the inherent blurring in PET scans. First, we applied a 3D reconstruction technique to integrate 346 individual slices into a single volumetric image, enabling simultaneous visualization of Aβ signals that would otherwise be fragmented across slices. Second, we utilized artificial intelligence technology, specifically the convolutional neural networks (CNNs), to suppress the background noise and enhance Aβ signal clarity in the PET scan. This AI-driven enhancement remarkably improved the visualization of characteristic spatial patterns, allowing for more accurate interpretation of signal distribution. If the PET signal were primarily driven by insoluble plaques, we would anticipate disorganized and heterogeneous imaging patterns, reflecting the sporadic nature of plaque deposition as typically observed in the histological sections [7,8,9,14]. In contrast, if the signal predominantly reflects soluble Aβ species distributed within structured non-CNS fluid compartments, the imaging should reveal organized and continuous spatial patterns. Third, we registered Aβ PET signals with MRI to assess their anatomical location. To minimize bias, we included both cognitively unimpaired (CU) and AD individuals in this retrospective analysis.

Following the background introduction in Section 1, the remainder of this paper is organized as follows: Section 2 outlines the Materials and Methods, detailing the procedures for data acquisition, imaging reconstruction, AI-assisted signal enhancement, and PET signal registration with MRI. Section 3 presents the Results, elucidating the spatial distribution and anatomical location of Aβ signals. In Section 4, we provide a Discussion of the findings in the context of amyloid clearance mechanisms and compartmental theory. Finally, Section 5 concludes the paper by summarizing the key insights and proposing future directions for research. Overall, we demonstrate that Aβ distribution in PET imaging consistently forms distinct and organized patterns across both CU and AD groups. These findings suggest that PET imaging may reflect not only amyloid burden but also aspects of soluble Aβ species involved in clearance dynamics, offering new insights into the interpretation of PET signals.

## 2. Materials and Methods

### 2.1. Study Design and Participants

Data used in the preparation of this article were obtained from the Alzheimer’s Disease Neuroimaging Initiative (ADNI) database (adni.loni.usc.edu). The ADNI was launched in 2003 as a public-private partnership, led by Principal Investigator Michael W. Weiner, MD. The original goal of ADNI was to test whether serial MRI, PET, other biological markers, and clinical and neuropsychological assessment can be combined to measure the progression of mild cognitive impairment (MCI) and early AD. For up-to-date information, see adni.loni.usc.edu. The current retrospective imaging study included CU individuals and patients diagnosed with AD. According to protocols at https://adni.loni.usc.edu/data-samples/adni-data/neuroimaging/pet (accessed on 1 May 2025–31 July 2025), there were no specific dietary restrictions for participants. Individuals received 370 MBq (or 10 mCi ± 10%). PET scan began 50 min post-injection. As shown in Table 1, a total of 12 subjects (6 CU and 6 AD) were selected for analysis based on image quality and completeness of anatomical coverage. All imaging data were anonymized and processed in accordance with institutional guidelines for data privacy and ethical research conduct.

### 2.2. PET Imaging Acquisition

Each scan consisted of sequential axial slices, acquired from the inferior to superior direction, with a slice thickness of approximately 2.0 mm. A total of 90 axial slices were obtained per subject. Coronal (128 slices) and sagittal (128 slices) views were generated during post-processing to provide comprehensive anatomical coverage. Raw PET images represented Aβ concentrations using grayscale pixel values ranging from 0 (black; no Aβ) to 255 (white; highest Aβ concentration). These images were downloaded as compressed ZIP files, securely stored on encrypted hard drives, and used as the basis for 3D reconstruction and subsequent analysis.

### 2.3. Reconstruction

The reconstruction was performed using 3D Slicer version 5.6.2, an open-source medical image computing platform available at https://www.slicer.org (accessed on 1 May 2025–31 July 2025), PET imaging data, provided in DICOM format, were imported into the software environment. Volume rendering was conducted using the Volume Rendering module within 3D Slicer, which enables real-time visualization of volumetric data. This module was used to reconstruct individual 2D axial slices into a continuous 3D volumetric image, preserving spatial relationships and signal intensity across slices.

### 2.4. Image Processing and Aβ Signal Enhancement

To enhance signal fidelity and reduce spatial blurring in PET imaging, convolutional neural networks (CNNs) were employed to suppress noise caused by positron straying and to amplify signals proximal to radioligand binding sites. This preprocessing step significantly improved the clarity and interpretability of volumetric reconstructions, which can be conducted with embedded algorithms in the 3D Slicer module called Volume Rendering. The module, primarily for CT scans relied on X-ray absorption by different densities of tissues, was slightly modified in the present PET studies. Specifically, scalar opacity mapping was applied to classify image data into four biologically relevant categories based on intensity and visualization goals, as illustrated in Table 2. The purposes of processing were to eliminate background noise and non-signal regions from visualization, filter out low-level signals below the visualization threshold, highlight biologically relevant Aβ signals within regions of interest, and preserve visibility of high-intensity signals while minimizing oversaturation. These scalar assignments were applied using adjustable filters for both intensity (Hounsfield Unit or HU, ranging from −1000 to 99,999) and opacity (0 to 1). It is important to note that these values were empirically determined and may vary depending on the acquisition parameters and imaging conditions across different laboratories. As such, the assigned intensity and opacity values should be calibrated according to the characteristics of the raw PET data. To minimize rendering artifacts and ensure accurate visualization, the processed images were considered acceptable only when the reconstructed patterns corresponded to those observed in the raw PET images, even if the latter appeared blurred. This validation step ensures that the enhanced images remain biologically and anatomically consistent with the original data.

To further improve the visual contrast of Aβ signals against the background, scalar (S) and model (M) parameters were adjusted during volume rendering. These parameters alter the steepness and midpoint of the transition curve, respectively, allowing fine-tuning of how signal intensity is translated into visual opacity. Contrast enhancement was achieved by either increasing the scalar or model values from 0 to 1.00 or decreasing them from 1.00 to 0, depending on the desired visualization outcome. The underlying algorithm for contrast adjustment involved multiplication or division of voxel intensity values, which modulates the opacity gradient across the volume. In the present study, setting either the scalar (S) or model (M) parameter to 0.50 was sufficient to achieve a balanced contrast, enabling clear visualization of Aβ signal distribution while minimizing background interference. This adjustment allowed for effective differentiation between low-intensity noise and biologically relevant signal patterns.

In the final step of image processing, voxel intensities were visualized using scalar color mapping based on a defined intensity gradient within the regions of interest (ROIs). The full intensity range from Point 1 (threshold) to the upper bound defined at Point 2 (biologically relevant Aβ signal) was divided into three equal segments to facilitate color-based interpretation. The lower third of the intensity range was assigned a green color, representing low concentrations of Aβ. The middle third was visualized using a yellow to brown gradient, indicating intermediate Aβ levels. The upper third and saturated Aβ were mapped to red, highlighting regions with the highest Aβ concentrations.

### 2.5. ROI Cropping and Post-Processing

Due to opacity settings at 0.5 or higher, the reconstructed 3D volumetric images were not immediately suitable for direct analysis of ROIs, as signal overlap reduced interpretability. To address this limitation, the cropping tool embedded in 3D Slicer was used to isolate specific ROIs for focused analysis. Based on empirical observation, selecting three consecutive slices within the 3D volume was sufficient to resolve the spatial relationships among Aβ signals with adequate clarity. In the present study, ROIs were categorized into four anatomical regions based on Aβ signal intensity and distribution: (1) Cranial regions; (2) Extracranial regions at the superior skull; (3) Extracranial regions in the deep cervical area; and (4) Extracranial regions in the superficial cervical area. In cases where Aβ signal intensity remained too low for reliable visualization, Fiji ImageJ version 1 software was employed for post-processing. This included contrast enhancement and background suppression, particularly in regions with subtle or diffuse Aβ deposition. These additional steps improved signal discernibility and supported more accurate interpretation of low-intensity regions.

### 2.6. Co-Registration with MRI and Brain Anatomy

To anatomically localize PET signals and assess their spatial relationship to structural landmarks, co-registration with MRI scans from the same individuals was performed using 3D Slicer. Raw DICOM files from both PET and MRI modalities were imported into the Volume module. In the co-registration workflow, MRI images were designated as the anatomical reference (background), while PET images served as the overlay (foreground). Alignment and rotation of the PET volumes were conducted within the Transforms module to achieve spatial correspondence with the MRI data. This process enabled precise localization of extracranial Aβ deposits and their anatomical context, including the skull, cervical vertebrae, and lymphatic structures. Co-registration was performed across axial, coronal, and sagittal planes to ensure comprehensive spatial alignment and visualization.

The anatomical locations analyzed in this PET study were defined according to the standardized brain regions delineated in the Atlas of the Human Brain by Mai and colleagues [15]. The atlas provides brain sections, MRI images, and schematic diagrams. Our regional definitions and interpretations follow the nomenclature and spatial coordinates presented in the fourth edition of the atlas, ensuring anatomical precision and reproducibility. The atlas is also accessible online at http://atlas.thehumanbrain.info (accessed on 1 May 2025–31 July 2025).

### 2.7. Imaging Data Interpretation

Spatial patterns of Aβ distribution were assessed through intensity-based analysis across anatomical coordinates. PET images were examined in three orthogonal planes: the sagittal view (left-to-right or LR), the coronal view (anterior-to-posterior or AP), and the axial view (inferior-to-superior or IS). In addition to static plane analysis, 3D rotation was employed to facilitate panoramic and whole-volume visualization, allowing for comprehensive assessment of Aβ signal organization. Aβ signals were categorized based on color-coded intensity levels derived from scalar color mapping. Within each ROI, colored signals were labeled sequentially to facilitate spatial tracking and comparative analysis. Signal morphology was classified into three distinct patterns:

- Sporadic: Signals appearing as isolated, short-distance deposits.
- Canal-like: Signals extending continuously over long distances, suggestive of directional flow or anatomical pathways.
- Network-like: Signals forming interconnected structures, potentially indicative of coordinated distribution or clearance routes.

This classification enabled systematic interpretation of Aβ spatial organization and its potential anatomical and pathological relevance.

## 3. Results

### 3.1. Processing a 3D Volume and ROIs

**Figure 1:**
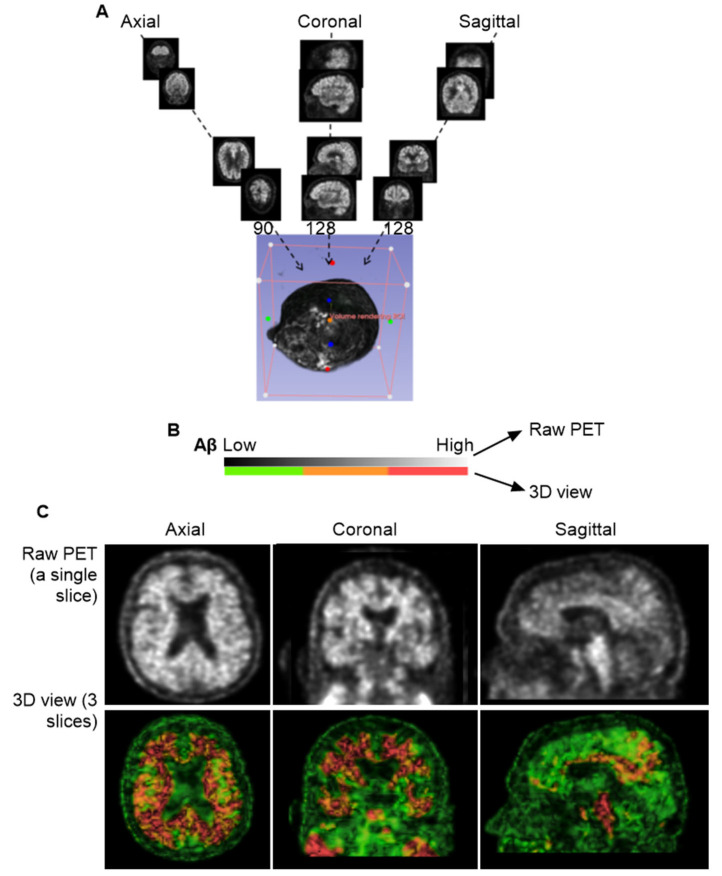
**Three-dimensional** Reconstruction for Florbetapir PET Imaging. (**A**), General workflow for generating a 3D volume from PET data. A total of 346 individual slices were synthesized into a single 3D image. The imaging data were obtained from the CU-1 individual (see Table 1 in Section 2) and used here as a representative demonstration. (**B**), Voxel-based color mapping of Aβ concentration following 3D reconstruction. Original PET grayscale values (ranging from 0 [black; no Aβ] to 255 [bright; highest Aβ]) were converted into voxel intensities and visualized using a color gradient: green (■) for the lowest Aβ levels, yellow-brown for intermediate levels (■), and red for the highest concentrations (■). (**C**), Comparison between raw PET imaging (top panel) and 3D reconstruction (bottom panel). The 3D reconstruction significantly improved image clarity, with optimal visualization achieved using three slices of 6.0 mm thickness.

The individual slice thickness in florbetapir PET scans is 2.0 mm, displaying as gray signals against the black background. At this resolution, the intensity of Aβ signals is often low and appears blurred to the human eye. To improve the analysis of the Aβ signal, we reconstructed these slices into a 3D volumetric image, enabling to gather more signals from regions of interest (ROIs) across multiple spatial dimensions. Figure 1A illustrates the transformation from 2D slices to a compact 3D volume encompassing the brain, skull, and portions of the cervical region. However, this initial 3D reconstruction was suboptimal for analysis and required further processing.

In the original PET images, Aβ concentrations were represented by grayscale pixel values ranging from 0 (black; no Aβ) to 255 (white; highest Aβ concentration). Following 3D reconstruction, these 2D pixels were converted into 3D voxels, each assigned to a color based on an arbitrary intensity scale (Figure 1B). In this study, voxel intensities were visualized using a color gradient: green for the lowest Aβ levels, yellow to brown for intermediate levels, and red for the highest concentrations.

To refine the imaging, we applied convolutional neural networks (CNNs) to filter out noise associated with positron straying far from radioligand binding sites, while enhancing signals proximal to ligand interactions. This CNN-based process significantly improved image clarity and reduced blurring. We found that using three consecutive slices was sufficient to produce a clear and interpretable image (Figure 1C). However, when more than ten slices were combined, the resulting visualization became overly complex, obscuring individual anatomical features. Interestingly, Aβ signals were detected not only within the brain parenchyma but also in peripheral regions, including the skull and cervical areas. Notably, Aβ concentrations appeared homogeneous in the skull but heterogeneous in the cervical region, suggesting a broader anatomical distribution and potentially dynamic clearance pathways beyond the central nervous system.

**Figure 2:**
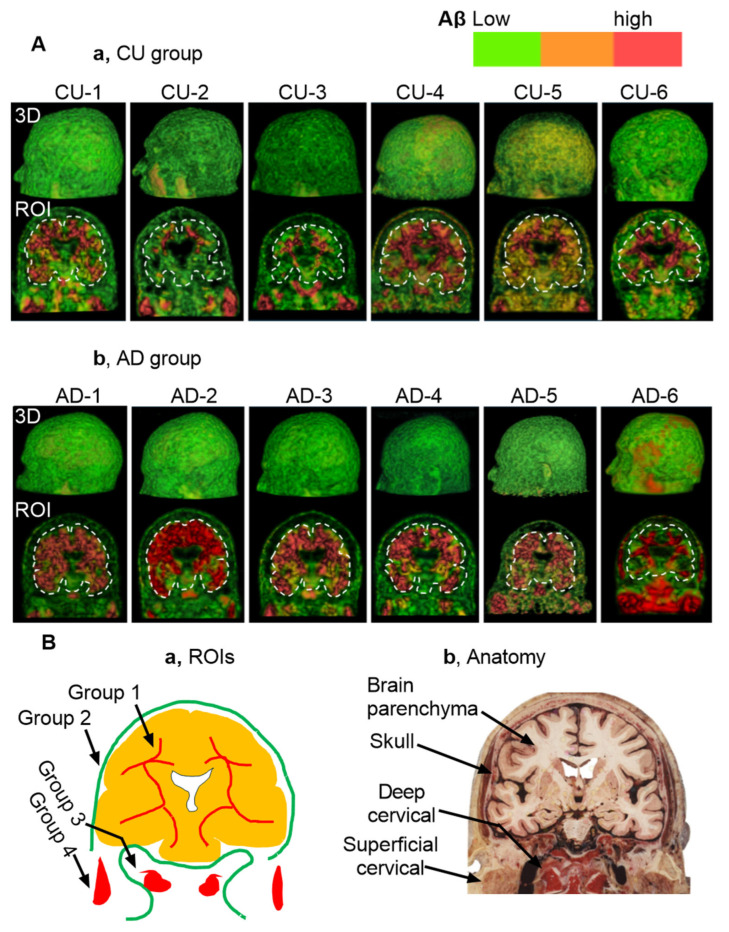
Static Views of 3D Reconstructions. Note that dynamic visualizations and clockwise rotation for 360° are available in Supplementary MP4 Video S1 and S2. Aβ levels are color-coded by intensity: green (■) indicates low expression, yellow or brown (■) moderate expression, and red (■) high expression. (**A**), 3D reconstructions from florbetapir PET scans in six CU individuals (**a**) and six individuals with AD (**b**). (**B**), Illustration of 4 regions of interest (ROIs) in the present study. (**a**,**b**) Anatomical mapping reveals that Aβ signals are heterogeneous in group 1 (i.e., brain parenchyma), group 3 (deep cervical areas), and group 4 (superficial cervical areas). In contrast, Aβ signals are low and homogeneous in the skull (group 2). Notably, both CU and AD groups exhibit a consistent spatial distribution pattern of amyloid beta, suggesting a shared anatomical clearance pathway. The anatomical illustration was excerpted from the Atlas of the Human Brain [15], and is used here to provide anatomical context for the described structure.

Figure 2A presents static pictures of 3D florbetapir PET scans from six cognitively unimpaired individuals (a) and six individuals with AD (b). MP4 videos for each subject provide dynamic visualization, included in the Supplementary Materials Videos S1 and S2. Aβ signal intensity was visualized using a color-coded scale: green for low expression, yellow to brown for moderate expression, and red for high expression. Since the color-coded scale is relevant to Aβ concentrations, it is likely that analysis of signaling colors could unearth Aβ kinetics in the normal tissues from the high to low concentrations. In the lymphoid tissues, however, the concentrations could be reversely from low to high concentrations due to autonomic regulation. Figure 2B depicts intensity mapping between Aβ signals and anatomies, revealed characteristic Aβ signaling patterns that can be grouped into four distinct ROIs: 1, cranial group; 2, extracranial group in the skull; 3, deep cervical group; 4, superficial cervical group (Figure 2B). Based on color-coded scales, the lowest Aβ signaling was in the skull, while the highest concentrations were observed along the brain ventricular walls, the occipital bones at the skull base, and the cervical lymph nodes (cLNs). Importantly, both CU and AD individuals exhibited consistent spatial distribution patterns, suggesting that the observed organization may reflect a shared anatomical pathway for Aβ clearance.

### 3.2. Group 1: Brain Parenchyma

**Figure 3:**
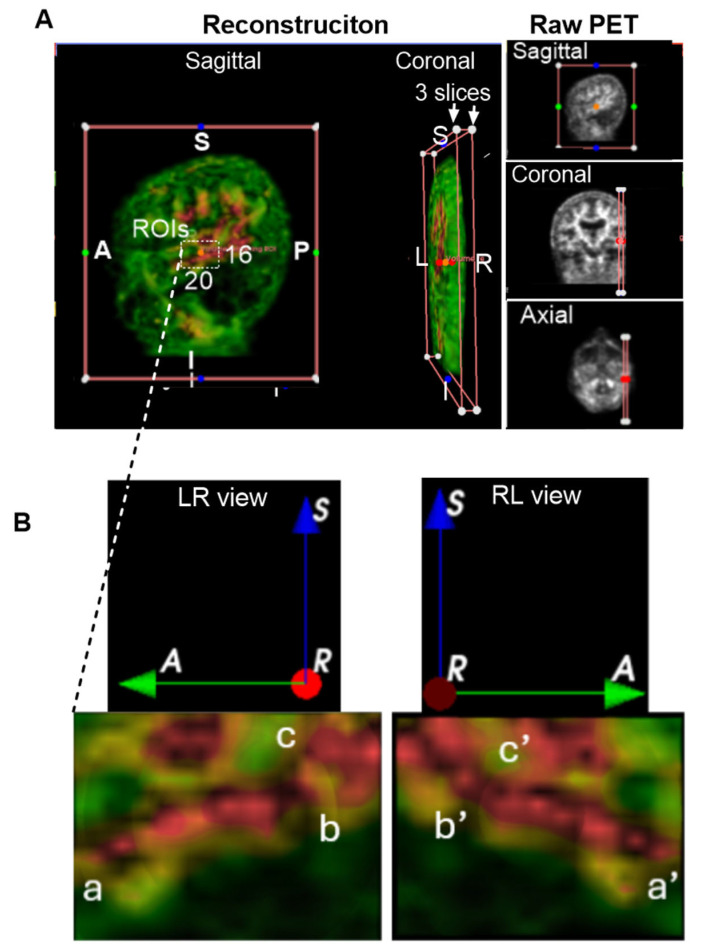
Group 1: Brain Parenchyma. Aβ levels are color-coded by intensity: green (■) indicates low expression, yellow or brown (■) moderate expression, and red (■) high expression. Anatomical orientation labels are shown: S, superior; I, inferior; A, anterior; P, posterior; R, right side of the subject; L, left side of the subject. Colored dots surrounding and inside the reconstructed images represent spatial reference points corresponding to individual slices from the raw PET data. These markers indicate the anatomical alignment and spatial distribution of signal hotspots across the imaging planes. (**A**), Representative 3D imaging obtained from the CU-6 individual. Dynamic rotation was captured as an MP4 video available in Supplementary Video S3. White boxes are indicative of regions of interest (ROIs) within the left temporal lobe. ROI dimensions included 20 AP slices, 3 LR slices, and 16 IS slices. A, anterior or frontal; P, posterior or rear; S, superior; and I, inferior. (**B**), Magnified view of the ROI, with three key points labeled as **a**, **b**, and **c**, for sequential clockwise rotations of the 3D structure to enhance spatial visualization. Corresponding rotated points **a′**, **b′**, and **c′** are shown, revealing network-like connectivity of Aβ signals within the temporal lobe.

We examined if Aβ signals within the brain parenchyma were the plaque-like pattern, which would be sporadic and randomly deposited in tissues, as shown in the postmodern studies [7]. To make a clearer interpretation, we selected a representative region characterized by a single red area (indicating high Aβ concentration) surrounded by green zones (indicating lower concentrations) for detailed visualization and description. Notably, similar patterns were consistently observed across all regions of interest (ROIs). The representative sample shown in Figure 3A was extracted from the left temporal lobe of the CU-6 individual. A dynamic 3D reconstruction of this region is available as an MP4 video in the Supplementary Materials Video S3.

A white-lined box in Figure 3A indicates the cropped ROI dimensions: 20 slices in the anteroposterior (AP) view, 3 slices in the left-right (LR) view, and 16 slices in the inferior-superior (IS) view. The resulting ROI is displayed in Figure 3B. In the LR view of Figure 3B, high-intensity Aβ signals are visibly organized into a network-like structure within the temporal lobe, labeled as points *a*, *b*, and *c*. To better visualize the 3D architecture, we performed clockwise rotations, revealing corresponding points *a′*, *b′*, and *c′* in the rotated RL views. These points appear to be continuously interconnected, forming a canal-like network structure within the temporal lobe. Although the precise diameter of this canal-like structure could not be measured, it is clearly larger than the microscopic scale typically associated with individual amyloid plaques. This observation suggests a potentially novel anatomical organization of Aβ distribution, distinct from traditional plaque-based models.

### 3.3. Group 2: Extracranial Aβ Signals in the Skull

**Figure 4:**
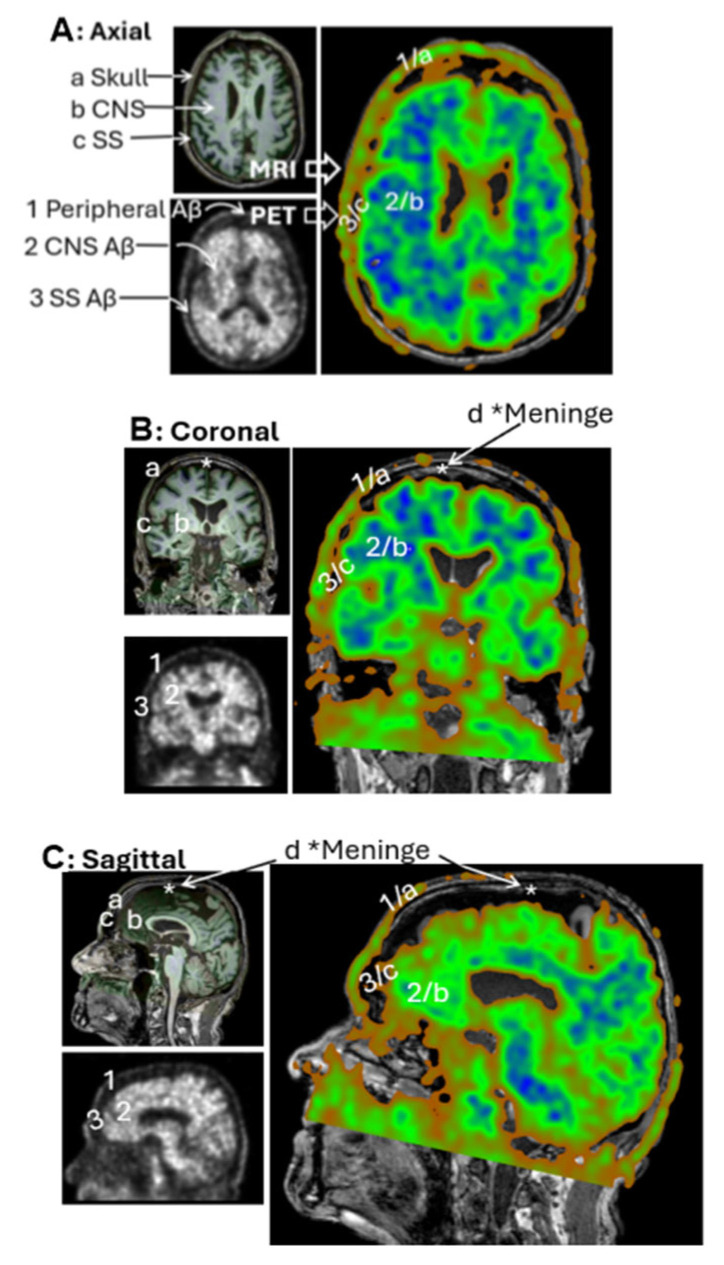
PET/MRI Coregistration. Representative MRI (left top panels) and PET scans (left bottom panels) from the CU-3 individual. Coregistration was conducted with 3D Slicer to set PET as the moving image (blue-green right panels) and MRI as the fixed reference (dark-gray right panels). Anatomical regions identified on MRI include **a**—Skull, **b**—Central nervous system (CNS), **c**—Subarachnoid space (SS), **d**—Meninges. Corresponding regions identified on PET include **1**—extracranial Aβ signal, **2**—CNS signal, **3**—SS signal. Subpanels: (**A**) Axial view, (**B**) Coronal view, (**C**) Sagittal view. Extracranial Aβ signals are spatially aligned with the skull, not the meningeal regions.

In raw PET imaging, Aβ signals are detectable in both the brain parenchyma and extracranial regions. However, extracranial signals have largely been overlooked and remain poorly characterized. To begin addressing this gap, we sought to understand the spatial relationship between extracranial Aβ deposits and anatomical structures of the head. This was achieved by co-registering PET images with MRI scans from the same individuals. Figure 4A presents an axial view of the co-registered images, revealing that extracranial Aβ deposits are spatially co-localized with the skull. This pattern of colocalization was consistently observed in the coronal (Figure 4B) and sagittal (Figure 4C) views. These findings suggest that PET signals in superior regions are anatomically associated with the skull, rather than with meningeal structures.

Next, we investigated whether Aβ signals in the superior skull region exhibited organized patterns or were randomly distributed, as typically seen with plaque deposition. We observed that Aβ concentrations in the skull were lower than those in the brain parenchyma, as indicated by green coloration on the intensity scale. To isolate Aβ signals in the skull, we carefully defined ROIs in the superior regions, explicitly excluding cranial Aβ signals from the axial, coronal, and sagittal planes of the brain parenchyma.

**Figure 5:**
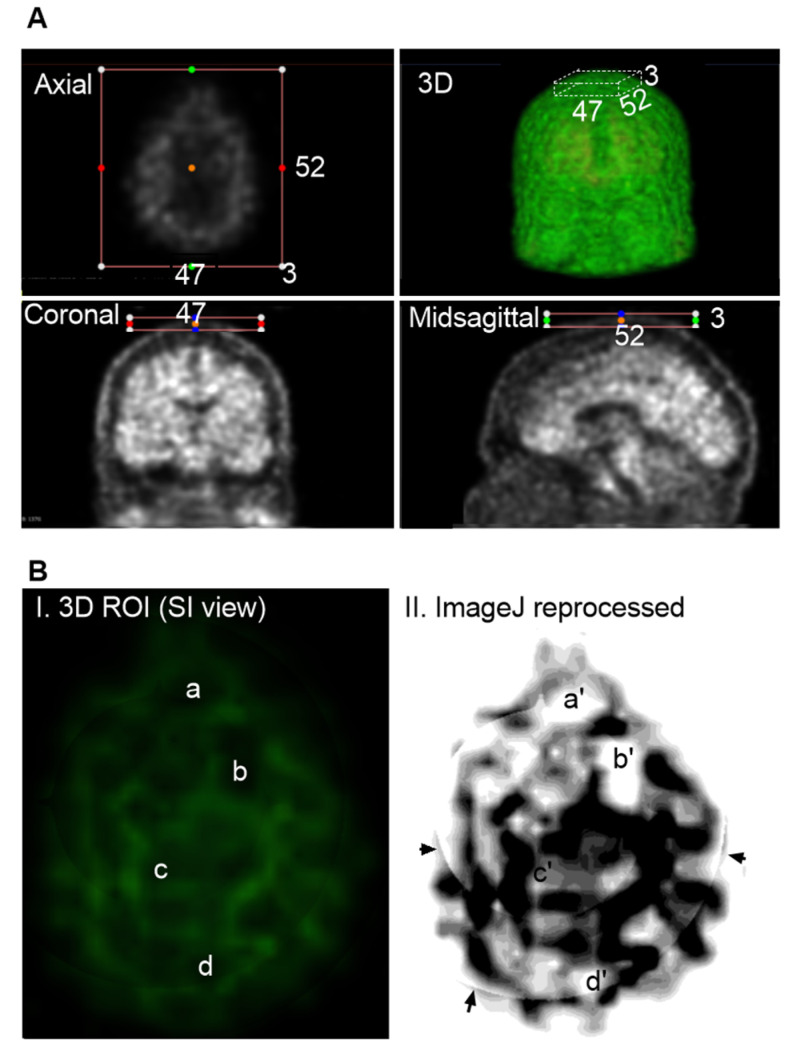
Group 2: Aβ Signals in the Skull. Representative imaging was based on AD-1 individual (002_S_0518). Raw PET scans displayed in axial, coronal, and midsagittal slices with overlaid colored dots. These dots, positioned within the surrounding boxes, serve as spatial reference markers, indicating the anatomical correspondence between individual 2D slices and the 3D reconstructed view. The color-coded markers help visualize the spatial distribution and alignment of signal hotspots across imaging planes, facilitating interpretation of regional tracer uptake patterns. (**A**), Cropping the regions of interest (ROIs) limited to extracranial areas, excluding any cranial Aβ signals. ROI dimensions were 52 AP slices, 47 RL slices, and 3 IS slices. (**B**), Visualization of Aβ distribution within the ROI. The green signals against a black background are indicative of Aβ on the skull. Regions without green signals are identified to have no Aβ signals, labeled as a, b, c and d (**I**). For a better view, 3D Aβ signals were reprocessed with Fiji ImageJ (**II**), corresponding regions as a′, b′, c′ and d′. In conclusion, Aβ signals are not evenly diffused or sporadically deposited as plaques. Instead, signals form organized network-like patterns across the superior skull. Arrows indicate regions that are not visible in the raw PET slices but appear in the reconstructed ImageJ, likely representing artifacts introduced during the integration of multiple slices.

Figure 5A illustrates a representative ROI selection strategy, with imaging dimensions of 52 slices in the anteroposterior (AP) direction, 47 slices in the right-left (RL) direction, and 3 slices in the inferior-superior (IS) direction. Figure 5B displays this extracranial ROI, finding that Aβ signals were spatially organized into distinct network-like patterns across the superior skull. Interestingly, these signals were not densely clustered as in the cranial regions. Several areas as labeled *a*, *b*, *c*, and *d* in the 3D visualization, showed no detectable Aβ signal. Due to the low concentration, the contrast against the black background was dim, making visualization challenging. To enhance clarity, the images were reprocessed using Fiji ImageJ (Figure 5B(II)), which improved the visibility of both the canal-like networks and the Aβ-free regions. In conclusion, low concentrations of Aβ were detected in the skull, forming organized canal-like networks that extended across the observed regions.

### 3.4. Group 3: Deep Cervical Areas

**Figure 6:**
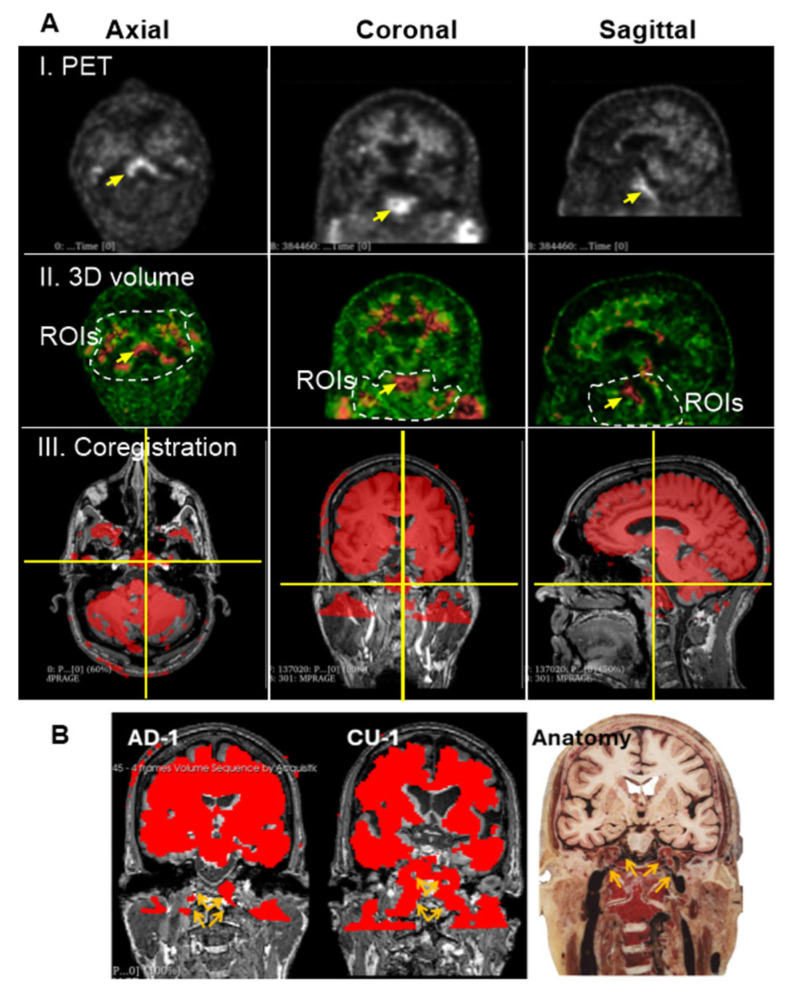
Group 3: the Deep Cervical Region. (**A**), High intensity of Aβ signals associated with the clivus bones. Prominent ROI, marked by yellow arrows, was consistently detected across PET scans of most individuals. Images displayed here are representatives of AD-1 (002_S_5018). Single arrow points to a region of high Aβ signal intensity located in the deep cervical area. (**I**), PET scan of a single slice at axial, coronal and sagittal planes. (**II**), 3D volume after reconstructing 3 individual slices for thickness. Dashed lines indicate the deep cervical areas. Aβ signal intensities range from green (low) to red (high). (**III**), Co-registration of PET/MRI reveals that this signal is anatomically aligned with the clivus, a bony structure at the base of the skull, anterior to the brainstem. The clivus is visualized at the intersection of the vertical and horizontal yellow lines across all three imaging planes. (**B**), PET signals are shown in red, representing Aβ tracer uptake. Images displayed here are representatives of CU-1 (002_S_0259) and AD-1 (002_S_5018). Brown arrays indicate the internal carotid plexus where Aβ signals in the place coregistered with MRIs of CU-1 and AD-1, respectively. The signals were associated with the internal carotid vessels entering the brain cavity. The anatomical illustration was excerpted from the Atlas of the Human Brain [15], and is used here to provide anatomical context for the described structure.

Unlike the skull, Aβ signal intensity in the deep cervical region is heterogeneous (Figure 2A), showing low in some ROIs and high in others. While low-intensity signals in this area also form canal-like networks, they appear more densely clustered than those observed in skull regions. High-intensity Aβ signals were localized to specific anatomical sites, as marked in Figure 6A(I,II). We hypothesized that these elevated signals in the deep cervical region may correspond to anatomical intersections involved in Aβ clearance pathways from the brain to peripheral lymphatic structures. To investigate this, we co-registered PET images with MRI scans from the same individuals to provide anatomical context for the high-intensity signals. This co-registration revealed that prominent Aβ signals were localized to the clivus of the occipital bone at the skull base (Figure 6A(III)). Additionally, high-intensity signals were observed along the internal carotid plexus (Figure 6B), suggesting that Aβ signals may extend beyond the cranial vault into structurally connected extracranial regions.

### 3.5. Group 4: Superficial Cervical Areas

**Figure 7:**
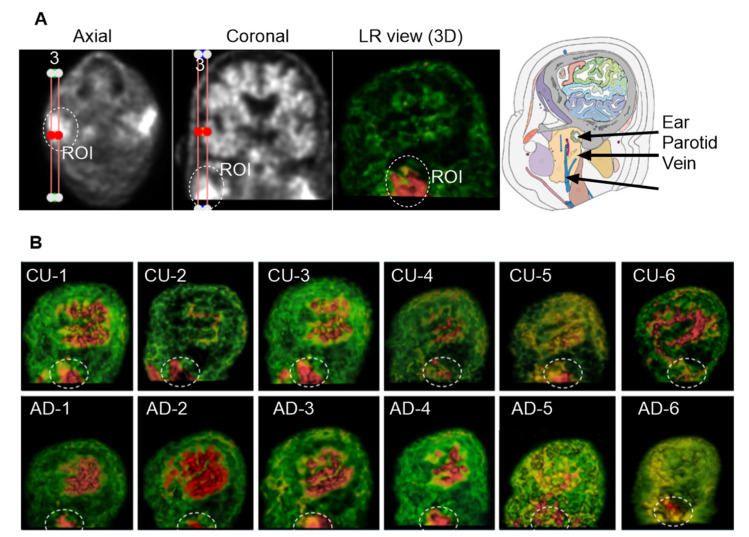
Group 4: the Superficial Cervical Region. The ROI was circled with dashed lines. (**A**), Three consecutive sagittal slices were reconstructed to enhance 3D visualization of the left-sided superficial cervical region. The highlighted ROI is located beneath the ear canal and adjacent to the parotid gland and jugular vein. (**B**), ROIs from six CU and six AD individuals. Although ROI size and shape vary across subjects, the region is consistently observed.

In the most inferior slices of the PET scans targeting the superficial cervical region, two highlighted areas were frequently observed on opposite sides of the superficial cervical zone. These regions appeared to be located beneath the ear canal, adjacent to the parotid gland and the external jugular vein. In this study, we focused on the left-sided highlighted region (Figure 7A). To enhance left–right spatial visualization, we reconstructed three consecutive sagittal slices into a 3D view. Figure 7B displays the corresponding ROIs from six CU individuals and six individuals with AD. Although the size and shape of the ROI varied among subjects, the region was consistently present across individuals and resembled the anatomical location of the superficial cLNs along the external jugular vein, specifically situated near the parotid glands. Due to limitations in imaging resolution, precise anatomical identification was not feasible at this stage.

## 4. Discussion

In the present study, we found that Aβ signals are organized into characteristic patterns in the brain. These patterns can be described from three key perspectives. First, Aβ appears to be arranged in long, canal-like lines with limited diameters, separated by intervening blank spaces. Notably, these structures were not observed within the four ventricular systems of the brain, supporting the interpretation that the observed signals originate from true Aβ signals in the brain parenchyma, rather than artifacts. Second, the same patterns were identified not only in the brain but also in the skull and cervical regions. However, the concentration and distribution of Aβ varied by location. We observed that the skull exhibited low and nearly homogeneous Aβ levels, while the brain parenchyma showed higher and more heterogeneous concentrations. In the cervical region, Aβ signals were also heterogeneous, with localized high concentrations in anatomically defined areas, likely corresponding to lymph nodes. Third, these organized patterns were consistently observed in both CU and AD individuals. Taken together, our AI-enhanced 3D analysis of PET imaging suggests that Aβ is primarily organized within canal-like networks that confine Aβ in a non-CNS fluid (NCF) compartment. This compartment is likely a metabolic clearance mechanisms that clear Aβ in the ISF compartment from the brain parenchyma and then to the skull lymphatic vessels and cervical lymph nodes (cLNs). These findings are consistent with previous reports that Aβ and tau clearance are likely through the peripheral lymphatic drainage [16,17,18].

The canal-like patterns have not been reported in the previous literature, likely due to limitations inherent in earlier methodologies. Investigations into Aβ distribution in the brain date back nearly a century, beginning with the application of Congo red staining by Paul Divry in 1927, who first described anomalous colors likely representing amyloid plaques under polarized light [19]. Congo red binds specifically to β-sheet-rich fibrillar Aβ, establishing a foundational method for identifying amyloid deposits in histological sections. Sensitivity to plaque detection was later improved using Thioflavin S in 1962, which enabled fluorescence-based visualization [20]. Radioligands such as Pittsburgh Compound B (PiB), florbetapir, and florbetazine could also be applied to postmortem tissues, followed by autoradiographic studies [21,22]. More recently, immunocytochemical techniques using antibodies against Aβ epitopes have shifted the focus from β-sheet structure to specific peptide sequences [7]. Despite the varieties, these studies have relied on in vitro postmortem brain tissue, where extensive washing steps during staining protocols likely remove soluble Aβ species, leaving only insoluble plaques visible. These plaques are found in the extracellular space, sporadically distributed mainly in the gray matter and fewer in the white matter [8,9,22], reinforcing the traditional view of Aβ localization in the extracellular space of the ISF compartment [see excellent reviews by [23,24]].

The first radioligand developed for Aβ PET imaging was ^11^C-Pittsburgh Compound B (PiB) in the early 2000s, followed by ^18^F-Florbetapir, ^18^F-Florbetaben, and ^18^F-Flutemetamol in the 2010s. In recent years, there has been a shift in terminology from “amyloid plaques” to “amyloid burden” when describing PET imaging results. This change reflects growing recognition that PET signals may represent not only insoluble plaques but also soluble Aβ species. Indeed, PET imaging typically shows widespread Aβ distribution over several millimeters, rather than sporadic microscopic plaques invisible to the human eye [e.g., <100 μm in diameter; see details in microplates [9]], suggesting that the PET signal may originate from different Aβ species than those observed in histological studies. This interpretation is supported by immunocytochemical evidence, demonstrating that antibodies against Aβ can detect plaques in tissue slices [7], but also soluble Aβ in biochemical assays such as Western blots [25]. Moreover, increasing concentrations of soluble Aβ have been shown to adopt β-sheet conformations [13], which are the binding targets of PET radioligands [6]. These findings provide evidence suggesting that PET imaging may more accurately reflect soluble β-sheet-rich Aβ species rather than insoluble plaques.

However, this alone does not explain why canal-like networks have not been observed in earlier PET studies. We propose two key reasons that have filled the gap between previous reports and present studies. First, previous PET studies typically analyzed individual slices [26], often around 2.0 mm in thickness, which may contain insufficient Aβ signal to reveal organized patterns. In contrast, our study synthesized all consecutive slices, enhancing signal strength and spatial continuity. Additionally, we applied color-coded voxel mapping, which is more visually sensitive than traditional grayscale (0–255 pixel intensity), allowing clearer detection of Aβ distribution. Second, image blurring has long been a challenge in PET imaging and yet has received limited investigation compared to quantitative metrics like SUVr [26] and CL values [2,3,27]. In the present study, we utilized 3D Slicer to reduce blurring and enhance the true signal. With the CNN-based algorithms, we were able to discern canal-like networks of Aβ signals that appear to reside in non-CNS fluid compartments, extending from the brain to the skull and cervical regions. Importantly, these patterns were observed in both AD and CU individuals, suggesting that Aβ may be physiologically confined prior to clearance. While the precise differences in the networks between AD and CU remain beyond the scope of this study, our findings highlight a novel anatomical context for Aβ distribution and clearance, warranting further investigation.

One remaining question is which anatomical structures within the brain could serve as compartments for Aβ accumulation and facilitate its metabolic clearance to peripheral systems. To fulfill this role, such structures must form distinct compartments that isolate Aβ from parenchymal cells, thereby minimizing neurotoxic interactions. Based on our anatomical observations, we identified five potential compartments where Aβ may reside: blood, CSF, ISF, glymphatic, and lymphatic systems. Among these, the ISF compartment is excluded from consideration due to its direct association with parenchymal cells. Similarly, the blood and CSF compartments lack distinct structural features in PET imaging and are unlikely to account for the organized networks observed.

The first plausible candidate for the anatomical basis of the networks observed in PET imaging is the glymphatic system, proposed in 2012 as a mechanism for Aβ clearance in the absence of identifiable lymphatic vessels within the brain. According to this hypothesis [28,29], the glymphatic pathway relies on astrocytes and their close association with small arteries and venules. Astrocytes are thought to line these vessels continuously, forming compartment-like structures that actively transport Aβ from the ISF into loosely connected perivascular spaces. Through pulsatile exchange between the glymphatic system and CSF, Aβ can be directed into the ventricular system and subarachnoid spaces. A second candidate, also involved in the glymphatic system, is the meningeal lymphatic vessels, which have been proposed to form part of a broader glymphatic–CSF–meningeal pathway [30,31]. This integrated system may represent a coordinated route for Aβ clearance from the brain to peripheral lymphatic structures, including the cLNs [32,33,34]. In our present study, we observed that the superior PET signals were spatially registered with the skull rather than the meninges on MRI, suggesting that meningeal lymphatics may play a less prominent role in the clearance process than previously thought. Whether astrocytes could serve as lining cells to form the canal-like structures observed in our 3D PET reconstructions remains an open question and warrants further investigation.

For many years, lymphatic vessels have been excluded from consideration of Aβ clearance in the brain. Historically, research on the lymphatic system has focused primarily on lab-friendly organs such as the gastrointestinal tract and mesenteries, while bones with dense and hard structures were long considered devoid of lymphatic structures. Recent advances in biotechnology have revealed the presence of lymphatic vessels within red bone marrow [35], suggesting that bones may also participate in waste-regulated clearance pathways. In our present study, the characteristic networks observed in the skull via PET imaging prompted us to consider their potential association with the peripheral lymphatic system, consistent with the previous reports [16,18,36]. Notably, the clivus at the occipital base of the skull frequently exhibited strong Aβ signals, further supporting this hypothesis that the red bone marrows in the skull sponges likely have lymphatic vessels that have homeostatic regulation of the Aβ clearance. Based on our imaging analysis, we propose two primary homeostatic pathways for Aβ clearance from the brain: one through skull-associated lymphatics leading to the superficial cLNs, and another through the internal carotid plexus toward deeper lymphatic drainage points. A deeper understanding of this anatomical clearance routes may offer novel therapeutic strategies to alleviate Aβ accumulation in the brain and improve treatment outcomes for neurodegenerative diseases such as Alzheimer’s.

## 5. Conclusions

In conclusion, the present study employed 3D reconstruction of florbetapir PET imaging combined with CNN-based filtration and enhancement to reveal Aβ signals throughout the brain and head regions in both CU and AD individuals. Our findings suggest that Aβ detected by PET is not primarily in the form of insoluble plaques within the ISF, as traditionally believed, but rather exists as highly concentrated soluble Aβ species confined within non-CNS compartments. These solubles appear poised for dynamic clearance via peripheral lymphatic pathways, including those associated with the skull and cLNs. Physiologically, this interpretation may help resolve the longstanding puzzle of how Aβ can be safely contained without exerting toxic effects on brain parenchymal cells.

However, several limitations of the current study must be acknowledged, particularly those related to the use of imaging algorithms and AI employed. First, we were unable to differentiate compartmental Aβ signal variations between CU and AD individuals, likely due to constraints inherent in the imaging capabilities of 3D Slicer. The algorithms employed were originally optimized for CT imaging, relying on X-ray absorption differences across tissue densities. Scalar X levels (Points 0–3) serve as proxies for tissue intensity, which are proportional to Hounsfield Units (HU)—with air defined as −1000 HU and water as 0 HU [37]. Consequently, there is a pressing need for PET-customized imaging algorithms specifically designed to quantify Aβ concentrations based on the positron emission intensity of florbetapir binding in PET data. Second, the AI-enhanced clarity of 3D imaging remains to be standardized. The key parameters, such as intensity, opacity, and scalar values, require further optimization to ensure consistent and reproducible visualization across datasets. Third, the molecular and cellular basis of the non-CNS compartments observed in the brain remains poorly understood. The structural and functional characteristics of these pathways are unresolved, limiting our ability to fully interpret their role in Aβ clearance. While our findings offer a promising direction for reinterpreting Aβ PET imaging and investigating novel clearance mechanisms, they should be viewed as a starting point for further exploration rather than a definitive conclusion.

## Figures and Tables

**Table 1:** Physical profiles of participants included in the present study.

Participants	IDA #	Age	Sex	Weight (kg)
CU-1	002_S_0295	90.1	M	73.0
CU-2	027_S_0074	83.4	M	80.0
CU-3	027_S_0118	86.3	M	110.6
CU-4	027_S_0120	83.7	M	70.8
CU-5	114_S_0173	79.4	M	75.7
CU-6	114_S_0416	87.3	F	41.3
	Mean ± SE	85.0 ± 22.1		75.2 ± 3.71
AD-1	002_S_5018	73.4	M	76.2
AD-2	027_S_4801	77.9	M	62.3
AD-3	027_S_4802	83.4	M	71.5
AD-4	027_S_4938	71.1	M	92.4
AD-5	114_S_4379	88.3	F	73.8
AD-6	114_S_6039	56.2	M	104.3
	Mean ± SE	75.1 ± 11.2		80.1 ± 15.4
	Compared to the CU, the unpaired *t*-test	*p* > 0.05		*p* > 0.05

The ‘#’ symbol represents the IDA search identifier assigned during the pooling of PET data https://ida.loni.usc.edu/login.jsp, accessed on 23 January 2024.

**Table 2:** Data filtration in 3D construction.

	Point	Intensity (X)	Opacity (O)	Scalar (S)
Background	0	−1000–0	0	0
Threshold	1	50–200	0	0
Aβ signals	2	800–2000	0.5–1.0	0.5
Saturated signals	3	2000–5000	0.5–1.0	0

## Data Availability

Data sharing is available at https://adni.loni.usc.edu/ (accessed on the 1 May 2025–31 July 2025).

## Supplementary Materials

The following supporting information can be downloaded at: https://www.mdpi.com/article/10.3390/cells14221754/s1. Video S1: Dynamic visualization of 3D reconstructions for six cognitively unimpaired (CU) individuals, corresponding to the static images shown in Figure 2A (CU group: CU1, CU2, CU3, CU4, CU5, and CU6). The region of interest (ROI) is rotated clockwise through 360°, providing a full spatial perspective of tracer distribution. Video S2: Dynamic visualization of 3D reconstructions for six individuals diagnosed with Alzheimer’s disease (AD), corresponding to the static images shown in Figure 2A (AD group: AD1, AD2, AD3, AD4, AD5, and AD6). The ROI is rotated clockwise through 360°, highlighting spatial patterns of amyloid beta accumulation. Video S3: Dynamic visualization and 360° clockwise rotation of the 3D reconstructed image displayed in Figure 3, illustrating detailed anatomical and tracer distribution features.

## Abbreviations

The following abbreviations are used in this manuscript:

PET	Positron emission tomography
3D	Three dimensions
IDA	Imaging and Data Archive
CU	Cognitive unimpaired
AD	Alzheimer’s disease
AI	Artificial intelligent
CL	Centiloid
CNNs	Convolutional neural networks
K_d_	Constant dissociation rate
ROI	Region of interest
SUVr	Standardized uptake value ratio
LR	Left-to-right
AP	Anterior-to-posterior
IS	Inferior-to-superior
cLNs	Cervical lymph nodes
